# Daily Consumption of Coffee and Eating Bread at Breakfast Time Is Associated with Lower Visceral Adipose Tissue and with Lower Prevalence of Both Visceral Obesity and Metabolic Syndrome in Japanese Populations: A Cross-Sectional Study

**DOI:** 10.3390/nu12103090

**Published:** 2020-10-11

**Authors:** Teruhide Koyama, Mizuho Maekawa, Etsuko Ozaki, Nagato Kuriyama, Ritei Uehara

**Affiliations:** Department of Epidemiology for Community Health and Medicine, Kyoto Prefectural University of Medicine, 465 Kajii-cho, Kamigyo-ku, Kyoto 602-8566, Japan; mizuho92@koto.kpu-m.ac.jp (M.M.); ozaki@koto.kpu-m.ac.jp (E.O.); nkuriyam@koto.kpu-m.ac.jp (N.K.); ruehara@koto.kpu-m.ac.jp (R.U.)

**Keywords:** coffee, green tea, visceral adipose tissue, metabolic syndrome

## Abstract

Background: The study aimed to investigate the association between daily consumption of coffee or green tea, with and without habitual bread consumption for breakfast, and components and prevalence of metabolic syndrome in Japanese populations. Methods: The study population consisted of 3539 participants (1239 males and 2300 females). Odds ratios (ORs) and 95% confidence intervals (CIs) were calculated using logistic regression analyses to evaluate the associations of daily coffee and green tea consumption with the prevalence of obesity, visceral obesity, and metabolic syndrome. Results: Coffee consumption was associated with significantly lower proportions of visceral obesity (OR: 0.746, CI: 0.588–0.947) and metabolic syndrome (OR: 0.706, CI: 0.565–0.882). On the other hand, green tea was not associated with visceral obesity (OR: 1.105, CI: 0.885–1.380) or metabolic syndrome (OR: 0.980, CI: 0.796–1.206). The combination of daily drinking coffee and eating bread at breakfast time was associated with significantly lower proportions of obesity (OR: 0.613, CI: 0.500–0.751) (*p* = 0.911 for interaction), visceral obesity (OR: 0.549, CI: 0.425–0.710) (*p* = 0.991 for interaction), and metabolic syndrome (OR: 0.586, CI: 0.464–0.741) (*p* = 0.792 for interaction). Conclusion: Coffee consumption was significantly associated with lower visceral adipose tissue and lower proportions of visceral obesity, but the same was not true for green tea consumption. Furthermore, in combination with coffee consumption, the addition of eating bread at breakfast time significantly lowered proportions of visceral obesity and metabolic syndrome, although there was no interaction between coffee and bread.

## 1. Introduction

A healthy diet helps to prevent chronic conditions such as cardiovascular disease, obesity, and metabolic syndrome [1]. The totality of one’s diet (that is, the combinations and amount of foods and nutrients consumed) is a crucial determinant of overall health. Therefore, experts emphasize food-based recommendations for the maintenance of healthy dietary patterns. However, epidemiological research has traditionally focused on individual isolated nutrients, which can result in erroneous conclusions [2]. For these reasons, the simple, evidence-based selection of a diet is an easy-to-understand and meaningful index for the maintenance of health in the general population.

Recently, one study reported that daily consumption of coffee or green tea was inversely associated with body composition and cardiovascular parameters in middle-aged Japanese women [3]. Coffee is considered one of the most widely consumed beverages worldwide, and several studies have associated coffee consumption with lower risks of metabolic syndrome [4] and obesity [5]. Green tea is also a popular beverage, particularly in Asian populations, and several reviews have demonstrated the potential role of green tea in the prevention of metabolic syndrome [6] and obesity [7]. Moreover, dietary patterns greatly influence beverage choices. The Mediterranean diet as a model of healthy eating may help to prevent weight gain and cardiovascular disease [8]. Bread is often eaten at breakfast with coffee and is part of the traditional Mediterranean diet; however, it has also been believed to induce obesity [9].

The present study aimed to investigate the association between consumption of coffee or green tea, with or without habitual bread consumption for breakfast, on components and prevalence of metabolic syndrome in Japanese populations.

## 2. Material and Methods

### 2.1. Study Population and Design

We previously published a cohort study known as the J-MICC study (Japan Multi-Institutional Collaborative Cohort Study) [10]. The present cross-sectional study included data from individuals who were enrolled in the J-MICC study’s second survey in the Kyoto field from 2013 to 2017 [11]. Of 3913 total participants, 374 were excluded due to missing data values. Thus, the study population ultimately consisted of 3539 participants (1239 males and 2300 females). All participants were recruited within the framework of the J-MICC study’s second survey in the Kyoto area. Written informed consent was obtained from all participants before participating, and the study protocol was approved by the Institutional Ethics Committee of the Kyoto Prefectural University of Medicine (approval number: RBMR-E-36-8 at 2013) and was conducted in accordance with the Declaration of Helsinki.

### 2.2. Data Collection and Measurements

All participants underwent a routine health check-up which included anthropometry (weight, height, waist circumference (WC)), blood pressure, measurement of visceral adipose tissue, and venous blood sampling. We also checked blood sampling time, postprandial time, and if there were any unanswered questionnaires. We used a standardized, self-administered questionnaire to acquire information about the participants’ consumption of coffee and green tea, consumption of bread at breakfast, smoking status, current drinking habits, medication use, average daily physical activity time, average daily sleeping time (hour/day), and type and frequency of leisure activities.

Information regarding coffee and green tea consumption was obtained in terms of frequency from eight selections (never, 1–3 times/month, 1–2 times/week, 3–4 times/week, 5–6 times/week, 1 time/day, 2 times/day, and ≥3 times/day). These eight selections were combined into four categories: less than daily, 1 time/day, 2 times/day, and ≥3 times/day. Habits regarding the consumption of bread at breakfast were divided into six categories from “rarely” to “every day”.

To quantify the patients’ smoking habits, the Brinkman index (a method commonly used to measure exposure to tobacco smoke) was calculated by multiplying the number of cigarettes smoked per day by the number of smoking years. To quantify alcohol consumption, we determined the alcoholic content of beverages consumed as well as the number of beverages consumed per day; these values were subsequently converted into the Japanese sake unit “gou” (180 mL), where 1 gou was equivalent to 23 g of ethanol. To evaluate medication regimens, we asked participants if they were taking medication for hypertension, dyslipidemia, or diabetes at least once a week. Physical activity was quantified using a format similar to the short format of the International Physical Activity Questionnaire (IPAQ) [12] and was assessed in terms of metabolic equivalents (METs) as previously reported [13,14]. In brief, METs (in terms of hours per day of leisure-time activity) were estimated by multiplying the associated MET intensity by the time spent each day for each reported activity.

Body mass index (BMI) was estimated as weight divided by the height squared (kg/m^2^). The cutoff point for obesity was set at a BMI of 25 kg/m^2^ based on the definition of obesity in Japan [15]. According to Japanese criteria, metabolic syndrome was defined as a male or a female having a WC of ≥85 cm or ≥90 cm, respectively, in addition to two or more of the following: lipid abnormality (a triglyceride level ≥ 150 mg/dL, a high-density lipoprotein cholesterol (HDL-C) level ≤ 40 mg/dL, or the use of lipid-modifying drugs); elevated blood pressure (an systolic blood pressure (SBP) level ≥ 130 mmHg, a diastolic blood pressure (DBP) level ≥ 85 mmHg, or the use of antihypertensive drugs); elevated blood glucose (a HbA1c level of ≥5.6%, or the use of drugs for diabetes) [16].

Visceral adipose tissue (VAT) was measured with a bioelectrical impedance analysis device (DUALSCAN, Omron Healthcare Co. Ltd., Kyoto, Japan). One previous report demonstrated that the intra-abdominal fat area measurements using DUALSCAN correlated well with intra-abdominal fat area measurements obtained via computed tomography in obese patients (*r* = 0.821, *p* < 0.0001) [17]. Visceral obesity was defined as a VAT measurement of ≥100 cm^2^.

### 2.3. Statistical Analysis

Continuous variables are expressed as means ± standard deviations, and categorical variables are expressed as sums and percentages. The significance of the univariate correlations between coffee and green tea consumption and other variables, including VAT, was assessed in accordance with Spearman’s rank correlation coefficient. Multiple regression analysis was also performed to compare continuous variables. VAT and BMI were used as dependent variables, and daily consumption frequencies (less than daily, 1 time/day, 2 times/day, and ≥3 times/day) of coffee and green tea were used as independent variables. All results were expressed after adjustment for confounders, including sex, age, Brinkman index, daily alcohol consumption, sleeping time, METs, and medication for hypertension, dyslipidemia, or diabetes. Odds ratios (ORs) and 95% confidence intervals (CIs) were calculated using logistic regression analyses to evaluate the associations of daily coffee and green tea consumption with the prevalence of obesity, visceral obesity, and metabolic syndrome. Information regarding coffee and green tea consumption was ultimately combined into two categories: “less than daily” and “one or more times daily”. Consumption of coffee or green tea less than once daily was used as a reference. The effect of breakfast time bread consumption was examined using logistic regression analysis, with the “less than daily” consumption category used as a reference. Two-way analysis of variance (ANOVA) with an interaction model was used for statistical analysis to clarify the interaction of bread with coffee and green tea and *p*-values for the interaction between bread and beverage consumption of coffee and green tea for components and prevalence of metabolic syndrome were calculated by multiple logistic regression analysis. A *p*-value of ≤0.05 was considered statistically significant. We analyzed all data using SPSS software (version 25, IBM Japan, Tokyo, Japan).

## 3. Results

Table 1 shows the characteristics of all participants. Approximately 28.0% and 50.6% of the participants, respectively, drank coffee and green tea less than once daily. Table 2 shows the correlations between each beverage consumption category (less than daily, 1 time/day, 2 times/day, and ≥3 times/day) and each anthropometric, lifestyle, or cardiometabolic parameter (BMI, SBP, DBP, triglycerides, total cholesterol, HDL-C, low-density lipoprotein cholesterol (LDL-C), HbA1c, METs, WC, VAT, Brinkman index, alcohol consumption, and sleep time). Table 2 describes how each of these parameters correlate with each category of beverage consumption per day. Coffee consumption was negatively correlated with triglyceride and VAT levels. In contrast, green tea consumption was positively correlated with triglyceride and showed no association with VAT.

Table 3 shows the adjusted multiple linear regressions between both beverage consumption categories and BMI and VAT in order to perform an analysis of the dose response of coffee and green tea consumption. An adjustment was carried out for the nine confounders. The multiple regression analysis showed that only coffee consumption was significantly associated with VAT (Beta = −1.652, *p* < 0.001). On the other hand, green tea consumption was not associated with BMI or VAT.

The multivariate-adjusted ORs for obesity, visceral obesity, and metabolic syndrome according to daily consumption of coffee and green tea are shown in Table 4. For the logistic regression analysis, consumption of coffee or green tea less than once daily was used as the reference. Dependent variables were consumption more than once per day and then adjusted for nine confounders. Neither coffee nor green tea consumption was associated with obesity. Coffee consumption was associated with significantly lower proportions of visceral obesity (OR: 0.746, CI: 0.588–0.947) and metabolic syndrome (OR: 0.706, CI: 0.565–0.882). On the other hand, green tea consumption was not associated with visceral obesity (OR: 1.105, CI: 0.885–1.380) or metabolic syndrome (OR: 0.980, CI: 0.796–1.206).

Next, we examined the effects of a combination of breakfast bread consumption and coffee and green tea consumption. The combination of drinking coffee more than once per day and eating bread at breakfast time was associated with significantly lower proportions of all dependent variables: obesity (OR: 0.613, CI: 0.500–0.751) (*p* = 0.911 for interaction), visceral obesity (OR: 0.549, CI: 0.425–0.710) (*p* = 0.991 for interaction), and metabolic syndrome (OR: 0.586, CI: 0.464–0.741) (*p* = 0.792 for interaction). The combination of drinking green tea more than once per day and eating bread at breakfast time was associated with significantly lower proportions of obesity (OR: 0.645, CI: 0.504–0.825) (*p* = 0.832 for interaction) and metabolic syndrome (OR: 0.659, CI: 0.499–0.870) (*p* = 0.850 for interaction). The results of two-way ANOVA also showed that the interaction of bread consumption at breakfast time with coffee and green tea consumption had no significant effect on VAT (*p* = 0.236 and *p* = 0.985, respectively). To clarify the interaction between breakfast time bread consumption and obesity, visceral obesity, and metabolic syndrome, we examined the effect on each independent variable according to whether coffee and green tea were consumed in Supplemental Table S1. Bread consumption at breakfast time was not associated with visceral obesity and metabolic syndrome when coffee consumption was less than 1 time/day.

## 4. Discussion

Coffee consumption is known to lower the risks of metabolic and cardiovascular conditions [4,5,18]. However, studies regarding the relationship between daily consumption of this beverage and VAT remain scarce, as VAT measurement requires diagnostic imaging. The results of the present study confirmed that each cup increment in daily coffee consumption was associated with a 1.652 cm^2^ lower VAT. Daily coffee consumption was associated with a 25.4% decrease in visceral obesity and a 29.4% decrease in metabolic syndrome. Furthermore, in addition to consumption of coffee at least once per day, the eating of bread at breakfast time was associated with a 45.1% decrease in visceral obesity and a 41.4% decrease in metabolic syndrome, although there was no interaction between coffee and bread.

In contrast, daily green tea consumption showed no significant association with lower visceral obesity or metabolic syndrome. Eating bread at breakfast time slightly enhanced the reduction effects of green tea on obesity and metabolic syndrome. This study suggests that the simple dietary habits of consuming coffee daily and eating bread at breakfast time significantly decrease VAT and are associated with lower visceral obesity and lower prevalence of metabolic syndrome. This study is the first to report a relationship between daily coffee consumption and visceral fat levels in a large population.

The anti-obesity effects of coffee and green tea have been shown in many basic molecular studies. Coffee has more than 1000 components, including caffeine and polyphenols such as chlorogenic acid. Green tea also contains various components, including caffeine and catechins such as epigallocatechin-3-gallate (EGCG). Caffeine, found in both coffee and green tea, has been determined to influence the energy balance in the body by increasing energy expenditure and decreasing energy intake [19]. Therefore, it has potential to be useful as a regulator of body weight. Furthermore, previous research has related the role of chlorogenic acid with glucose and lipid metabolism [20]. A randomized controlled trial showed that consumption of high-chlorogenic-acid coffee for 12 weeks by obese adults reduced VAT, BMI and waist circumference [21]. In summary, participants who exhibited higher levels of coffee consumption were associated with better energy balance and lower visceral fat accumulation.

Alternatively, there have been many studies on the association between green tea (which also contains caffeine) and metabolism; consumption of the catechins found in green tea reduced abdominal fat accumulation [22,23] and improved biomarkers of metabolic syndrome [22,23]. However, a Cochrane review proved that the weight loss in adults who consumed green tea was not statistically significant and was not likely to be clinically important [24]. Although data from many laboratories indicate that components such as catechin and caffeine in green tea may be involved in metabolic syndrome inhibition, further in vivo studies are needed to investigate the temporal and dose-response relationships governing individual mechanisms due to many unclear in vivo functions [25,26]. In fact, some studies reported that coffee but not green tea consumption was inversely associated with metabolic syndrome in Japanese [27,28]. EGCG may have more of an effect on inflammation than on metabolism [29]. From an endocrinology viewpoint, plasma adiponectin levels are associated with multiple risk factors related to visceral fat accumulation [30]. Although several findings have suggested a positive association between coffee consumption and adiponectin level, other studies have shown contradictory results regarding green tea consumption and adiponectin level [31]. In non-obese people, coffee may have an effect on visceral fat loss, but green tea may affect inflammation more than it affects metabolism.

The addition of bread to breakfast was associated with less visceral fat and obesity when compared to coffee alone. In addition, Supplementary Table S1 analyzed by classification shows that bread consumption is not associated with reductions in visceral obesity or improved parameters of metabolic syndrome in participants who consume coffee less than 1 time/day. This result may be due to increased coffee consumption by drinking coffee when eating bread. However, there is no interaction between bread consumption at breakfast time and consumption of coffee and green tea for VAT from the results of two-way ANOVA and multiple logistic regression analysis. The consumption of bread and coffee was independent, and each consumption was associated with prevalence of visceral obesity and metabolic syndrome. Reducing consumption of white bread, not whole-grain bread, is associated with less weight gain and less abdominal fat gain [32]. In the present study, it is unknown whether participants consumed white bread or whole-grain bread. The type of bread consumed (either white or whole grain) may have affected the response on visceral fat accumulation and metabolic syndrome. As bread is often eaten at breakfast with coffee, the combination of meals may have some effect, and additional studies should analyze employing the effect of the bread-making method on body weight and metabolic regulation. It is difficult to predict why breakfast-time bread consumption reduces the prevalence of visceral obesity and metabolic syndrome, but it is likely that bread choice is a factor in overall coffee consumption. In any case, simple, evidence-based diet selection is an easy-to-understand and meaningful index for the promotion of public health in the general population.

This study has several limitations that require consideration. First, this study implemented a cross-sectional design and measured daily beverage and bread intake only by questionnaire; therefore, actual consumption volumes, total energy intake, sugar in green tea or coffee or other food consumption, type of bread consumption, and presence of caffeine were not measured. Second, we had no measurements of caffeine, catechins or polyphenols from participants’ blood samples which may have helped explain some changes in cardiometabolic parameters. Third, the cohort studied only included healthy Japanese participants. Further studies to address the question of causality and other populations are necessary to validate our findings. A strength of this study is that it measured VAT in large numbers. Accurate measurement of VAT is difficult, and few reports have measured VAT on such a large scale.

## 5. Conclusions

The results of this study demonstrated that coffee consumption was significantly associated with reduced VAT and lower proportions of visceral obesity, but the same was not true for green tea consumption. Furthermore, in combination with the consumption of coffee at least once per day, the added consumption of eating bread at breakfast time significantly lowered proportions of visceral obesity and metabolic syndrome, although there was no interaction between coffee and bread.

## Figures and Tables

**Table 1:** Characteristics of participants.

	All = 3539		Male = 1239		Female = 2300		*p*-Value
	Mean	SD	Mean	SD	Mean	SD	
Age (year)	57.6	10.	58.6	10.1	57.0	9.9	<0.001
BMI (kg/m^2^)	22.2	3.24	23.5	2.98	21.6	3.16	<0.001
SBP (mmHg)	129	19.2	135	18.0	126	19.0	<0.001
DBP (mmHg)	79.2	11.3	83.1	10.8	77.1	11.1	<0.001
TG (mg/dL)	100	72.3	122	87.7	88.4	59.1	<0.001
Total cholesterol (mg/dL)	217	36.0	205	31.9	223	36.5	<0.001
HDL-C (mg/dL)	69.9	17.1	61.1	15.0	74.6	16.2	<0.001
LDL-C (mg/dL)	126	31.0	122	29.3	128	31.6	<0.001
HbA1c (%)	5.58	0.46	5.64	0.55	5.55	0.41	<0.001
METs (hours/day)	14.5	10.3	13.9	10.6	14.9	10.2	0.006
WC (cm)	80.9	9.26	84.9	8.35	78.7	9.00	<0.001
VAT (cm^2^)	61.5	32.7	79.5	35.4	51.8	26.4	<0.001
Brinkman index	212	408	489	541	63.7	189	<0.001
Alcohol (g/day)	11.9	20.8	22.0	27.9	6.49	12.8	<0.001
Sleep time (hour)	6.40	0.98	6.49	1.00	6.35	0.96	<0.001
	n	%	n	%	n	%	
Obesity (BMI ≥ 25)	656	25.2	353	28.5	303	13.2	<0.001
Visceral obesity (VAT ≥ 100)	436	12.3	327	26.4	109	4.7	<0.001
Metabolic syndrome	557	15.7	398	32.1	159	6.9	<0.001
Medication	n	%	n	%	n	%	
Hypertension	590	16.7	306	24.7	284	12.3	<0.001
Dyslipidemia	540	15.3	212	17.1	328	14.3	0.014
Diabetes	112	3.2	73	5.9	39	1.7	<0.001
Coffee consumption	n	%	n	%	n	%	
Less than daily	991	28.0	354	28.6	637	27.7	<0.001
1 time/day	999	28.2	315	25.4	684	29.7	
2 times/day	891	25.2	290	23.4	601	26.1	
Over 3 times/day	658	18.6	280	22.6	378	16.4	
Green tea consumption	n	%	n	%	n	%	
Less than daily	1793	50.6	661	53.3	1132	49.2	<0.001
1 time/day	536	15.1	206	16.6	330	14.3	
2 times/day	431	12.2	146	11.8	285	12.4	
Over 3 times/day	779	22.0	226	18.2	553	24.0	
Breakfast bread consumption							
every day	1472	41.6	479	38.7	993	43.2	0.005

BMI, body mass index; SBP, systolic blood pressure; DBP, diastolic blood pressure; TG, triglycerides; HDL-C, high density lipoprotein cholesterol; LDL-C, low-density lipoprotein cholesterol; WC, waist circumference; VAT, visceral adipose tissue; METs, metabolic equivalents; HbA1c, glycated hemoglobin.

**Table 2:** Correlations between the consumption of coffee and green tea and each parameter.

	Coffee		Green Tea	
	Coefficient	*p*-Value	Coefficient	*p*-Value
BMI	0.023	0.172	−0.011	0.499
SBP	−0.026	0.128	0.061	<0.001
DBP	−0.009	0.579	0.018	0.283
Triglycerides	−0.006	0.001	0.034	0.043
Total cholesterol	0.002	0.925	0.037	0.029
LDL-C	0.023	0.170	−0.002	0.898
HDL-C	0.012	0.477	0.033	0.048
HbA1c	−0.020	0.239	0.065	<0.001
METs	−0.004	0.794	0.021	0.218
WC (cm)	−0.014	0.422	0.000	0.983
VAT	−0.049	0.003	0.005	0.752
Brinkman index	0.109	<0.001	−0.075	<0.001
Alcohol (g/day)	0.032	0.059	−0.090	<0.001
sleep time	−0.053	0.002	0.024	0.161

Beverage consumption category (less than daily, 1 time/day, 2 times/day, and ≥3 times/day). BMI, body mass index; WC, waist circumference; VAT, visceral adipose tissue; METs, metabolic equivalents.

**Table 3:** Multiple linear regressions between each categorized consumption of coffee and green tea and BMI and VAT.

	Coffee		Green Tea	
	Beta	*p*-Value	Beta	*p*-Value
BMI	−0.004	0.928	0.029	0.489
VAT	−1.652	<0.001	0.312	0.428

Adjusted for age, sex, daily alcohol drinking, brinkman index, sleeping time, METs, drug treatment for hypertension, dyslipidemia and diabetes. Beverage consumption category (less than daily, 1 time/day, 2 times/day, and ≥3 times/day). BMI, body mass index; VAT, visceral adipose tissue; METs, metabolic equivalents.

**Table 4:** Comparison of the association of consumption of coffee and green tea and bread at breakfast time with visceral obesity and metabolic syndrome.

	Coffee More Than 1 Time/Day		Green Tea More Than 1 Time/Day		Coffee More Than 1 Time/Day and Eating Bread at Breakfast Time (*n* = 1172)		Green Tea More Than 1 Time/Day and Eating Bread at Breakfast Time (*n* = 730)	
	OR	95% CI	OR	95% CI	OR	95% CI	OR	95% CI
Obesity (BMI ≥ 25)	0.869	0.715–1.055	0.983	0.821–1.176	0.613	0.500–0.751	0.645	0.504–0.825
Visceral obesity (VAT ≥ 100)	0.746	0.588–0.947	1.105	0.885–1.380	0.549	0.425–0.710	0.778	0.578–1.047
Metabolic syndrome	0.706	0.565–0.882	0.980	0.796–1.206	0.586	0.464–0.741	0.659	0.499–0.870

Adjusted for age, sex, daily alcohol drinking, brinkman index, sleeping time, METs, drug treatment for hypertension, dyslipidemia and diabetes. OR, odds ratios; CI, confidence intervals.

## Supplementary Materials

The following are available online at https://www.mdpi.com/2072-6643/12/10/3090/s1, Table S1: Comparison of the association of consumption of bread at breakfast with visceral obesity and metabolic syndrome.
