# Supplementary material for: Daily Consumption of Coffee and Eating Bread at Breakfast Time Is Associated with Lower Visceral Adipose Tissue and with Lower Prevalence of Both Visceral Obesity and Metabolic Syndrome in Japanese Populations: A Cross-Sectional Study
Source: Nutrients. 2020 Oct 11;12(10):3090. doi: 10.3390/nu12103090 (PMC7599629; doi:10.3390/nu12103090)
Supplement: Supplementary file 1 [file nutrients-12-03090-s001.pdf]

Supplemental Table 1. Comparison of the association of consumption of bread at breakfast with visceral obesity and metabolic syndrome

	Coffee less than 1 time/day		Green tea less than 1 time/day		Coffee more than 1 time/day		Green tea more than 1 time/day	
	OR	95% CI	OR	95% CI	OR	95% CI	OR	95% CI
Obesity (BMI ≥ 25)	0.655	0.443-0.969	0.640	0.489-0.838	0.588	0.469-0.736	0.567	0.429-0.748
Visceral obesity (VAT ≥ 100)	1.130	0.718-1.778	0.602	0.428-0.846	0.549	0.413-0.730	0.700	0.500-0.979
Metabolic syndrome	0.770	0.500-1.187	0.621	0.454-0.849	0.614	0.472-0.798	0.621	0.453-0.850

Adjusted for age, sex, daily alcohol drinking, brinkman index, sleeping time, METs, drug treatment for hypertension, dyslipidemia and diabetes
